# Knowledge and awareness of warning signs about Lung cancer among Pharmacy and Nursing undergraduates in Riyadh, Saudi Arabia - an observational study

**DOI:** 10.7150/jca.89358

**Published:** 2023-10-09

**Authors:** Omaimah A. Qadhi, Alya Alghamdi, Dalal Alshael, Maha Fayez Alanazi, Wajid Syed, Ibrahim Nasser Alsulaihim, Mahmood Basil A. Al-Rawi

**Affiliations:** 1 Department of Medical-Surgical College of Nursing, King Saud University, Riyadh, 11451, Saudi Arabia.; 2 Department community and mental health, college of nursing, Riyadh, 11451, Saudi Arabia.; 3 Department of Nursing Administration & Education, College of Nursing, King Saud University, Riyadh, 11451, Saudi Arabia.; 4 Department of Clinical Pharmacy, College of Pharmacy, King Saud University, Riyadh, 11451, Saudi Arabia; 5 Department of Pharmacology and Toxicology, College of Pharmacy, King Saud University, Riyadh, 11451, Saudi Arabia.; 6 Department of Optometry, College of Applied Medical Sciences, King Saud University, Riyadh, 11451, Saudi Arabia.

**Keywords:** Lung cancer, Coughing, awareness, Risk factors, Treatment, Saudi Arabia.

## Abstract

**Background:** Cancer is becoming more common, regardless of gender or type. Cancer was determined to be the leading cause of death, with lung cancer (LC) patients having the highest rate of cancer-related deaths. The purpose of this study was to analyze undergraduates' knowledge and awareness of LC early warning signs in Riyadh, Saudi Arabia.

**Methods**: Between May and September 2022, a cross-sectional, prospective paper-based survey-type study was conducted among undergraduates (n=202) from the faculty of pharmacy and nursing at King Saud University (KSU) in Riyadh, Saudi Arabia. The data was gathered from third and fourth-year undergraduates. The statistical package for social science (SPSS Inc., Chicago, IL, U.S.) was used to perform the analysis.

**Results:** The mean age of the undergraduates was 22.47 ± 2.35(SD) years. Most of them were from nursing 54% (n=109), while 46% (n=93) belonged to a pharmacy. In terms of awareness of warning signs of lung cancer, 48.6% of the students believed that unexplained weight loss, followed by persistent chest infection (36.6%) and cough that does not go away easily (37.6%). Over 45.1 % of students opted that coughing up blood, pain during the cough (46.5%), and worsening or change in an existing cough (42.1%) were reported as a sign of LC. In this study, the overall good awareness score was 60(29.7%). The awareness was significantly associated with gender (*p* = 0.0001), the course of study (*p*=0.018), the educational level (*p* = 0.003), smoking cigarettes (*p* = 0.003), and chronic disease status (*p* = 0.0001).

**Conclusion:** Undergraduates attending university in this study indicated various levels of awareness of LC symptoms. The undergraduate's educational background, study program, and gender all greatly influence their level of awareness. It is necessary to inform future medical professionals about this growing condition.

## Introduction

Cancer is a term for a variety of medical disorders classified as malignant tumors or neoplasms 1-3. Cancer is a chronic disease characterized by uncontrolled or abnormal cell development and spread, which can rapidly spread to other parts of the body, resulting in hospitalization and death if not treated swiftly 1,2. Cancer is the leading cause of death, with lung cancer (LC) patients having a higher rate of cancer-related deaths. It was found that LC is the second most prevalent cancer diagnosed globally 3,4.

The American Cancer Society predicted that in 2022, an estimated 236,740 new cases of LC would be identified in the United States, with 130,180 people dying as a result of the condition 2. Furthermore, cancer-related mortality was more frequently recorded in poor and middle-income countries worldwide 5. A recent Epidemiological Analysis in Saudi Arabia discovered 4530 LC incidences between 2006 and 2016. In different locations of Saudi Arabia, the age-standardized incidence rate ranged from 1.2 to 12.3 per 100,000 cases among males. The LC incidence increased with age, with 23% reported in the age group of more than 75 years 6.

Alcohol consumption, air pollution and unhealthy eating habits are risk factors for cancer and other noncommunicable diseases even while exposure to multiple physical, chemical, and biological carcinogens was listed as the main cause of cancer and tobacco 1. Interestingly, smoking continues to be a major factor in almost 80% of LC fatalities in the US, both in terms of incidence and mortality rates 2. Due to the disease's subtle symptoms, which include a prolonged cough, bloody sputum, chest pain, a hoarse voice, deteriorating of breath, and recurrent pneumonia, is imperative to identify LC. 1,2

Nationally and internationally published literature revealed a lack of knowledge about LC among undergraduates. For instance, a previous study among health students in Saudi Arabia reported poor awareness of LC risk factors (76.6%) 7. It was evidenced that Students with greater awareness and knowledge express better Awareness towards LC screening 8. Similarly, data suggested that being female gender, pursuing medicine, and having the marital status of an undergraduate are associated with good knowledge and awareness of LC 8-10. Preliminary literature reported that the gap or lack of knowledge of LC among undergraduates both nationally and internationally can increase morbidity and mortality 7-10. There were lack of studies assessing the knowledge of LC among students in Saudi rabid. However, there were studies evaluated the clinical presentation of various other diseases among undergraduates in Saudi Arabia which includes diabetes 11, thyroid cancer 12, herbal 13, and Supplements^14^. Before graduation, having a thorough awareness and knowledge of various infections positively influences clinical practices in the future 11,12. Studies on LC awareness, among healthcare undergraduates, are rare in Saudi Arabia and other international countries. The comprehension of many elements of LC disease among senior pharmacy and nursing, students in Saudi Arabia has not been the subject of any research. Furthermore, undergraduates, especially pharmacists, nurses, and medical professionals, are on the first boundaries, ready to help patients as needed 11,12. Therefore, the purpose of this study was to assess the knowledge and awareness of healthcare students regarding LC in Saudi Arabia.

## Materials and Methods

### Study Design, Settings, Population

This cross-sectional questionnaire-based study was conducted among undergraduates studying at the faculty of pharmacy and nursing at KSU in Riyadh, Saudi Arabia, over two months September to October 2022. This study included undergraduates in their third to final year. Undergraduates were contacted directly and asked to complete the questionnaire only once to avoid duplicate responses. The study included those who showed an interest in filling out the questionnaire. The institutional review board of KSU College of Medicine, Riyadh, Saudi Arabia, reviewed and approved the study protocol before data collection (E-21-6371). Before data collection, students provided informed consent, assuring that their information would be kept private and used solely for study. Additionally, this study adhered to the Declaration of Helsinki's rules and took the necessary precautions to protect the welfare and rights of the human participants 15.

### Study Questionnaire, Data Collection, and Source

Following a review of the literature 15,16, the questionnaire was developed. This study's questionnaire has been divided into two groups. The first group has nine questions that analyze undergraduates' demographic features as well as clinical information such as disease history, smoking status, and shisha use. The second category contains questions about LC knowledge and awareness (14 items), which are rated on a 5-point Likert scale with '1' being strongly disagreed and '5' being strongly agreed. The questionnaire was created in English and then translated into Arabic with the assistance of a native Arabic speaker utilizing forward and reverse translation processes. The questionnaire was then evaluated in two steps. First, the initial draft was examined by a research expert in the associated field to confirm that the questionnaire's content and flow were correct. Second, a pilot study with a randomly selected sample of 30 students was undertaken to gather input. Students were approached at random for the pilot study. Students were told about the goal of the study before completing the surveys, and their participation was entirely optional. Cronbach's alpha was calculated to be 0.72 to establish dependability. The pilot study data was not included in the final analysis.

The final questionnaire was then distributed to undergraduates during break time at both the pharmacy and nursing colleges, with the assistance of a data collector. The data collection was followed by convenience sampling. Eligible undergraduates were invited to participate in the study by filling out a questionnaire. The data collectors included a senior researcher who was knowledgeable about how to recruit participants, collect data, and enable the survey's completion. Following previous research, scores were assigned to each of the scale's items by categorizing the undergraduate's responses as correct or incorrect 17-20. Similar to previous studies 17-20, correct responses were assigned a score of one, while incorrect responses were assigned a score of zero 17-20. Furthermore, responding with 'strongly agree' or 'agree' was considered a correct response, whereas responding with 'strongly disagree,' 'disagree,' or 'not sure' was considered an inaccurate answer. The scores were prepared for both knowledge of respiratory and non-respiratory symptoms. Similarly, the overall knowledge score was prepared by computing all items (item 14). Further, the overall score was divided into three levels poor (who scored >25 percentile), moderate between 26-50 percentile of the total score, and good knowledge score who scored above <50 percentile of the total score.

**Scheme 1:**
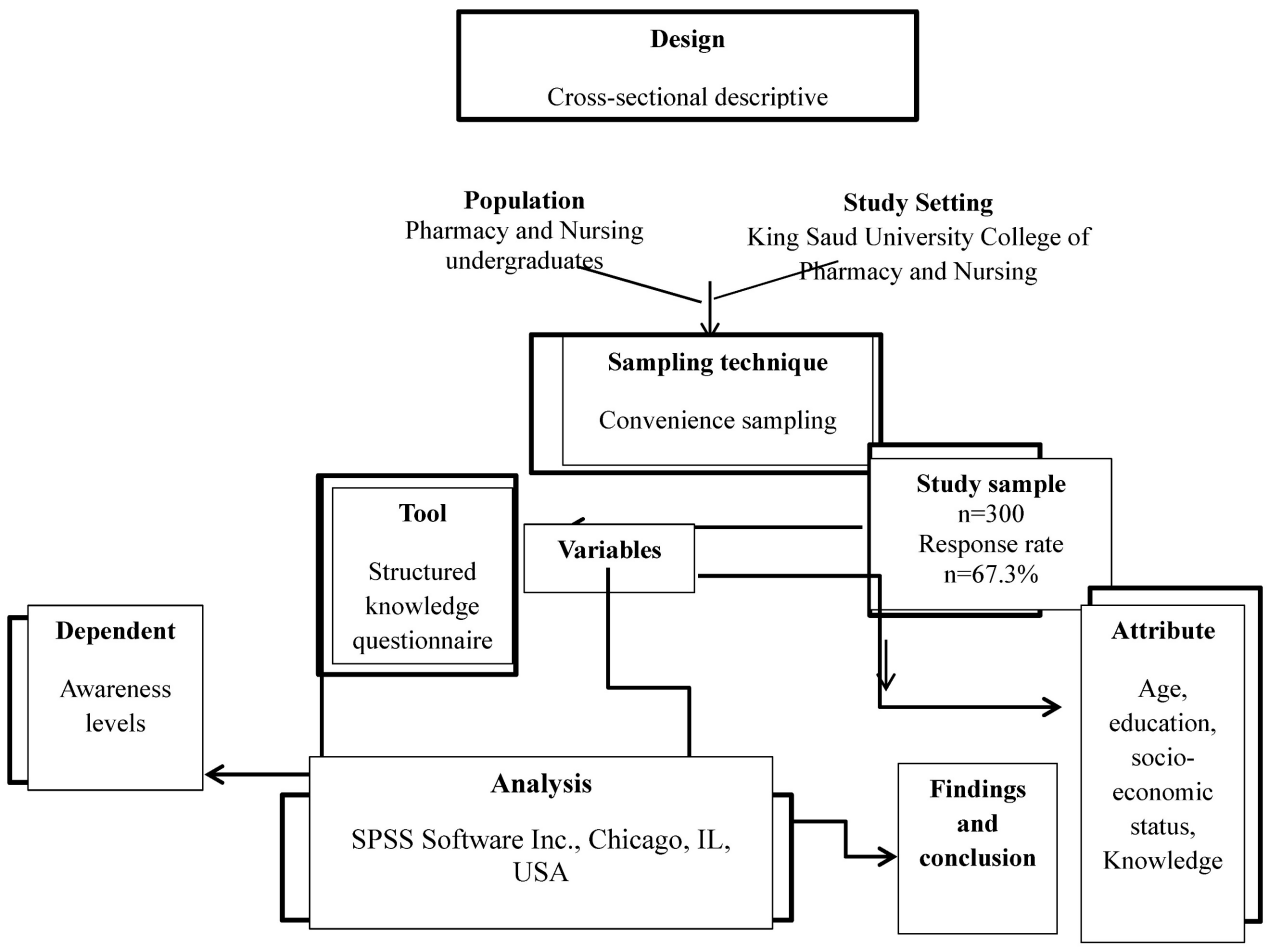
Flow chart of study design.

During the period of study, we found approximately 600 undergraduates from both colleges at the KSU campus were taking their courses. Similar to previous studies we computed the sample size 21-24 using an online sample size calculator with a 95% confidence level and a predetermined margin of error of 5%, as in prior investigations. Because we were unclear of the potential outcomes for each question, we assumed that the response distribution for each question would equal 50%. Although the predicted sample size was 235, we chose to survey at least 300 students to ensure more reliability. Scheme 1 describes the study's design and sample in detail.

### Data analysis

Frequencies (*n*) and percentages (%) were used to describe categorical variables, chi-square test was used to perform comparisons. The dependent variables were considered numeric variables, and the independent variables were categorical, so we used multiple linear regression analysis to compute the association. A P-value of < 0.05 was considered a statistically significant difference, and the data were analyzed using Statistical Package for Social Sciences version 26.0 (SPSS Inc., Chicago, IL, USA).

## Results

A total of 202 college students participated in this survey, giving a response rate of 67.3%(n=300). Of whom 78.7 % (n = 159) were males. The mean age of the undergraduates was 22.47 ± 2.35 years, with ages ranging between 18 to 25 years. Most of the students were from Nursing 54% (n=109), while 46% (n=93) were pharmacy students. More than half of the students were in the fifth academic year. The majority of the students were single (91.1%). While 176 (87.1%) of the studied individuals were free from chronic disease. The detailed descriptions of the respondents are given in Table 1.

**Figure 1:**
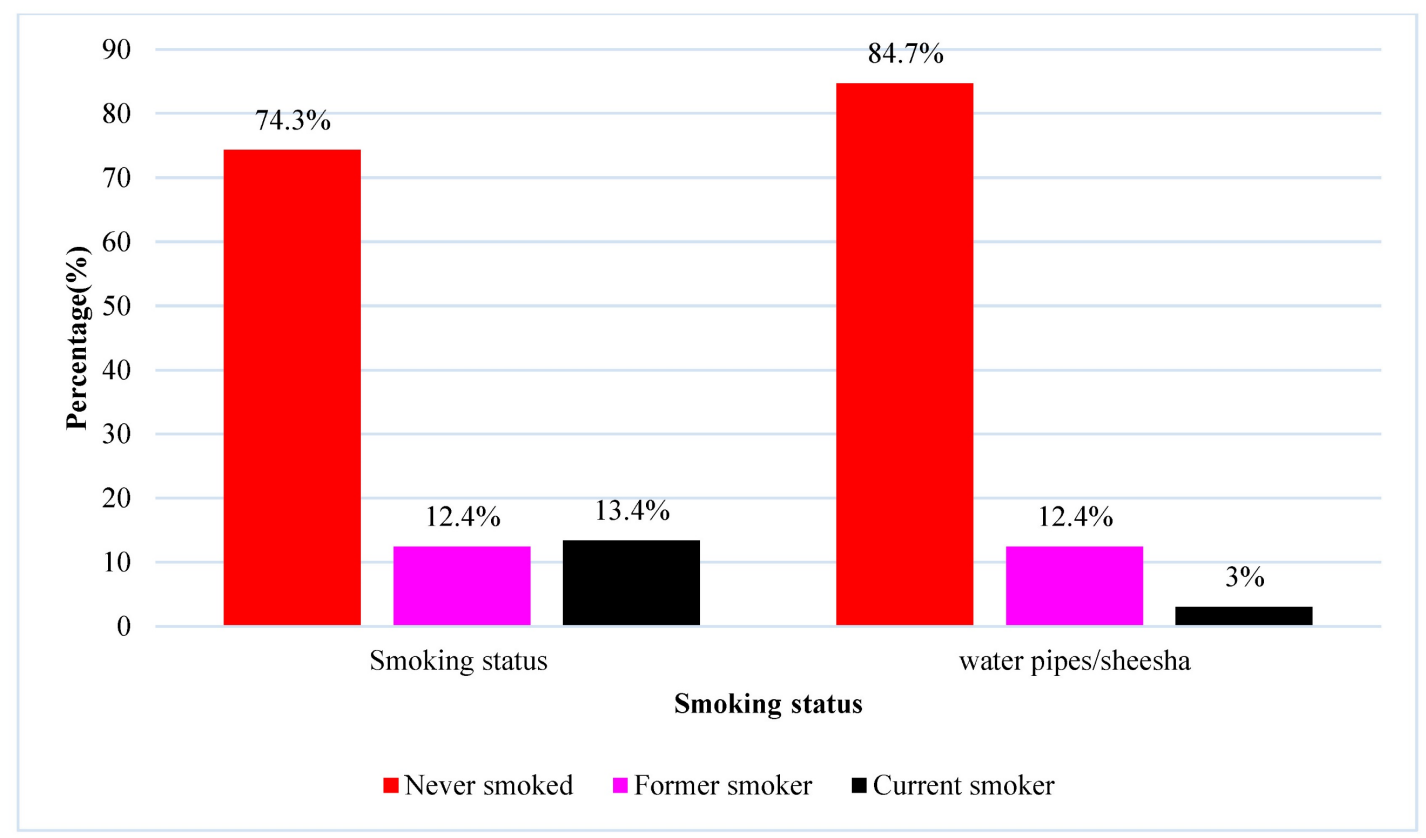
Smoking status of the undergraduates.

Regarding smoking status, 74.3%(n=150) of the students never smoked while 84.7%(n=171) students never smoked water pipes/shisha. Only 13.4%(n=27) and 3.0%(n=6) of the undergraduates smoked cigarettes and water pipes/shisha (Figure 1).

### Awareness of warning signs of lung cancer

In terms of awareness of warning signs of lung cancer, 48.6% of the students believed that unexplained weight loss was a sign of LC (2.98 ±1.46; Median (3.0), while 36.6% (Median 3.0; IQR (3)) of the students agreed that persistent (3 weeks or longer) chest infection (3.00 ± 1.32; IQR (2)). On the other hand, 37.6% (Median 3.0) of the students positively responded to a cough that does not go away for two or three weeks can lead to LC (3.08±1.34; IQR (2). Over 48 % (3.26+1.35) of students believed persistent shortness of breath was a sign of LC (Median 3.0; IQR (2). Furthermore, 46% of students reported tiredness or lack of energy, while 43.1% of them identified chest pain, and more than one-third of the students (35.1%) agreed that shoulder pain was the sign and symptom of LC (Table 2).

Over 45.1 % of students opted for coughing up blood, 46.5% pain during the cough, and 42.1% Worsening or change in an existing cough reported as a sign of LC. Slightly less than half reported an ache or pain when breathing, while more than one-third 37.6 % reported developing an unexplained loud, high-pitched sound when breathing is the possibility of lung cancer. Only (36.1%) believed that changes in the shape of your fingers or nails as one of the signs of lung cancer. Detailed information regarding signs and symptoms is described in Table 3.

**Figure 2:**
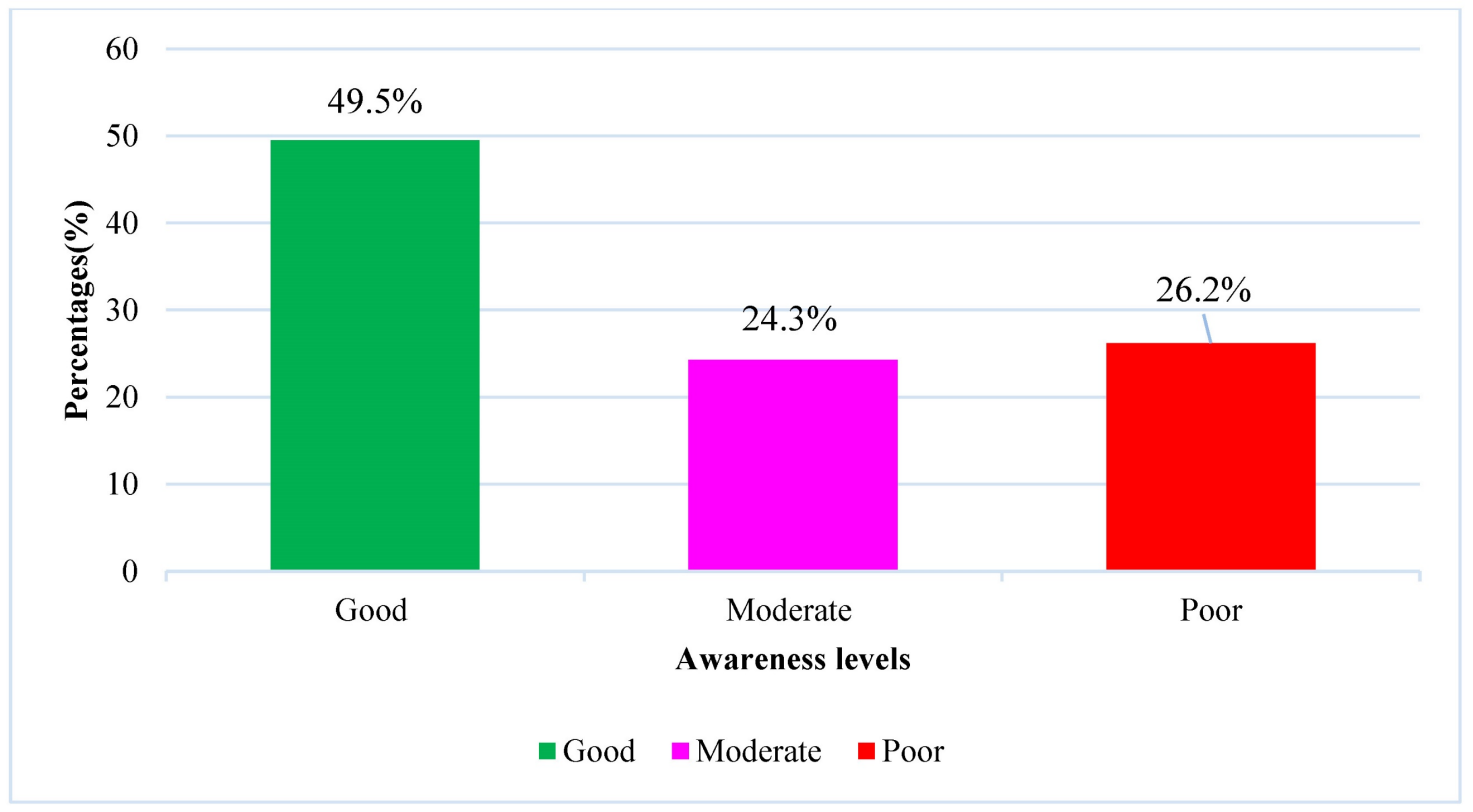
classification of total awareness levels.

In this study, the overall good awareness score was 60(29.7%), while 47(23.3%) undergraduates were found to have moderate awareness about lung cancer, while most of them 95(47%) found poor awareness of LC as shown in Figure 2. The awareness is significantly associated with gender (*p* = 0.0001), the course of study (*p*=0.018), the educational level of the undergraduates (*p* = 0.003), smoking cigarettes (*p* = 0.003), and chronic disease status (*p* = 0.0001). Table 4 also provides a full explanation of the relationship between undergraduate awareness levels and demographics.

A Multiple linear regression model was used to investigate the relationship between the knowledge score of LC and student demographics, with gender, age, education level, course of study, ever-smoked cigarettes, ever-smoked water pipes/shisha, and history of chronic disease considered explanatory variables and knowledge of awareness score as the dependent variable. There was a strong relationship between gender, marital status, and the prevalence of chronic disease in students with knowledge of LC awareness. The regression model results showed a significant correlation between knowledge of LC awareness and gender (p=0.008), marital status (p=0.001), and presence of chronic disease (p=0.002), as shown in Table (5).

## Discussion

Our study assessed Knowledge and awareness of cancer among health college students towards LC as well as the associated features. In general, studies investigating LC knowledge and awareness in prospective undergraduates, particularly in the contexts of pharmacy and nursing, resources were scarce. The goal of this study was to raise student awareness and knowledge since an early age-increasing trend of using nicotine in various forms (e-cigarettes and vaping) was a risk factor that was more prevalent among middle-aged undergraduates. In general, most undergraduates reported that tobacco smoking is a potential risk factor for lung cancer. Similar findings were reported by Al-Naggar in 2012 among university students, and Zainuddin and colleagues among undergraduate students, where the majority of the students agreed that smoking, air pollution, and occupational hazards were the most common risk factors for LC 8,9. In this study, the proportion of undergraduates who displayed smoking as the risk factor for LC was similar to the proportion who were aware of cancer or thyroid cancer and agreed that exposure to smoking also leads to the development of cancer 12. It is crucial to recognize risk factors and take action to reduce them, especially when it comes to smoking. Tobacco use should also be reduced by instilling fear, guilt, and denial in smokers about smoking-related illnesses like LC.

The proportion of undergraduates who displayed good levels of awareness of LC symptoms was 29.7%. The current findings show variation in awareness. The good awareness was significantly different concerning the course of study. For instance, pharmacy undergraduates reported good awareness (31.2%) in comparison to nursing undergraduates (28.4%). The levels of awareness in this study and the previous study varied. For instance, a previous survey conducted in 2022 by Elshami et al. revealed that 51.8% of people had an excellent understanding of the signs of lung cancer. Due to the different age groups of the undergraduates and study methodology 16. Additionally, pharmacy students participate in a variety of smoking cessation programs and receive additional instruction about the risks of smoking, which may account for the increased awareness of LC symptoms among them 25.

The smoking prevalence in this study is similar to earlier study among Saudi undergraduates (Alwhaibi et al., 2022), for instance, Alwhaibi and colleagues 2022 found that 20.3% of the undergraduates were smokers, in addition approximately 28% of the undergraduates smoke >10cigrates per day, surprisingly only 31.7% of the students received education on the dangers training 25. Which suggested that Saudi undergraduates were knowledgeable about the risk factors of lung cancer. Despite knowing the risk factors, in this study, one-quarter of the undergraduates were current and former smokers. This raised the interest in creating more awareness and knowledge about the harmful effects of smoking which may lead to several types of cancers, more particularly LC since undergraduates are future practitioners who are responsible for delivering health care to patients and the public. To raise risk awareness, alter behaviors, and promote early recognition and presentation, health policymakers in Saudi Arabia must concentrate on the identification of LC risk factors while implementing educational interventions and efficient tobacco control programs 12,13.

The study found that weightlessness was the most frequently reported non-respiratory LC symptom, followed by chronic fatigue or lack of energy, shoulder pain, loss of appetite, changes in the appearance of the fingers or nails, and the emergence of an unexplained loud, high-pitched sound when breathing. These findings were in line with those previously reported by Elshami et al in 2022 among Palestinians, who noted persistent fatigue or a lack of energy, followed by a decrease in appetite and weight loss as well as the development of an obtrusive loud, high-pitched sound when breathing 16. Regarding knowledge of respiratory symptoms, most undergraduates identified a cough that lasts for three weeks or more, a chest infection that has persisted for longer than three weeks, shortness of breath, chest pain, coughing up blood, achy or uncomfortable breathing, a painful cough, or a cough that is getting worse or changing. These findings are consistent with earlier findings among undergraduates by Zainuddin et al in 2017, who showed that the symptoms of LC patients included exhaustion, shortness of breath, wheezing, hemoptysis, chest discomfort, and recurring respiratory infections 8. Similar to Elshami et colleagues in 2022, Palestinians stated that LC symptoms included increasing or changing an existing cough, followed by coughing up blood persistent shortness of breath pains, or pain when breathing 16.

This might indicate a better understanding of respiratory problems. In this study, undergraduates might read about or talk about potential respiratory symptoms with less embarrassment. Health-related subjects about LC would also be of interest to both men and women and would be of greater appeal to students and the general public. Both industrialized and developing nations are seeing an increase in the risk factor for developing lung cancer. The high prevalence of smoking in the Saudi Arabian setting, particularly among men and women in their college years and maturity, may also have a role in the increased awareness of LC symptoms 25.

The current research found that both the undergraduate students' characteristics and their levels of awareness of LC varied significantly. The level of awareness is significantly associated with gender, educational discipline, education levels, smoking habits, and the presence of chronic diseases. Similar results were reported by Zainuddin et al. and colleagues, who discovered that socio-demographic characteristics such as age, year of study, and course type were substantially related to students' knowledge 8. Similar findings were made by Al-Naggar in his 2012 study, which discovered that students' understanding of LC is highly influenced by their marital status, their choice of teacher, and the semester they are in 9. Targeted awareness programs on lung cancer's detrimental effects and risk factors are crucial so that smokers are aware of their higher risk and can seek medical attention sooner if they exhibit any potential symptoms. Additionally, these campaigns may help dispel a frequent misconception among smokers that neglects the possibility of LC as a possible diagnosis and instead solely associates respiratory symptoms with smoking itself. It is crucial to address smokers' fear, self-blame, and denial about smoking-related disorders, including LC, when creating these programs.

A few limitations exist in our study. First, it is a descriptive observational study that aims to evaluate pharmacy and nursing students' knowledge of awareness of LC symptoms. Second, because the results were based on a self-administered assessment, biases like social desirability bias and recollection bias may have been more likely to exist. Thirdly, because the data came from just one Saudi Arabian university, they cannot be generalized to other universities or the entire world. Despite these drawbacks, a report of this kind is essential to identify any difficulties, if any, and then to further explore how they affect patients before presenting solutions. Having said that, our study is the first of its kind and will lay the groundwork for future research on this topic.

## Conclusion

In conclusion pharmacy and nursing undergraduates attending Saudi University in, Riyadh City indicated various levels of awareness of LC symptoms. The undergraduate's educational background, study program, and gender all greatly influence their level of awareness. The exposure to disease in particular is triggered by a lack of understanding and awareness of diseases in clinical settings, which could be interpreted adversely and potentially lower the quality of life for patients. More importantly, compared to both national and international research, LC prevalence was increasing, which may signal an increase in unfavorable effects. The current findings may help pharmacy and nursing faculties adapt their curricula to facilitate the creation of communications intended to raise awareness of lung cancer. As a result, we support the establishment of educational initiatives that inform students about the clinical and psychological impacts of LC to help them avoid difficulties and live healthy lifestyles.

## Figures and Tables

**Table 1:** Demographic characteristics of the study sample (n=202).

Demographics	Frequency(n)	Percentage (%)
**Gender**		
Male	159	78.7%
Female	43	21.3%
**Marital status**		
Single	184	91.1%
Married	16	7.9%
Divorced	2	1.0%
**Course of study**		
Pharmacy	93	46%
Nursing	109	54%
**Year of Study**		
Third	17	8.4%
Fourth	38	18.8%
Fifth	107	53%
Final	40	19.8%
**Do you have any chronic diseases?**		
Yes	26	12.9%
No	176	87.1%
**Risk factors for Lung cancer?**		
Smoking	178	88.1%
Air pollution	39	19.3%
Occupational exposure	25	12.3%
Exposure to others smoking	41	20.29%
**Do you know someone diagnosed with cancer?**		
Yes	54	26.7%
No	148	73.3%

**Table 2:** Frequencies of Undergraduates Toward Awareness of Warning Signs of LC (n=202).

Signs/symptom	Strongly agree n (%)	Agree n (%)	Neutral n (%)	Strongly Disagree n (%)	Disagree n (%)	Mean± (SD)
Unexplained weight loss	41(20.3)	37(18.3)	56(27.7)	55(27.2)	13(6.4)	2.98+(1.46) Median (3.0) IQR (3)
Persistent (3 weeks or longer) chest infection	35(17.3)	39(19.3)	54(26.7)	33(16.3)	41(20.3)	3.00±1.32 Median (3.0) IQR (2)
A cough that does not go away for two or three weeks	40(19.8)	36(17.8)	61(30.2)	34(16.8)	31(15.3)	3.08±1.34 Median (3.0) IQR (2)
Persistent shortness of breath	43(21.3)	54(26.7)	54(26.7)	36(17.8)	15(7.4)	3.26+1.35 Median (3.0) IQR (2)
Persistent tiredness or lack of energy	46(22.8)	47(23.3)	53(26.2)	25(12.4)	31(15.3)	3.28+1.31 Median (3.0) IQR (2)
Persistent chest pain	46(22.8)	41(20.3)	55(27.2)	32(15.8)	28(13.9)	3.20+1.36 Median (3.0) IQR (2)
Persistent shoulder pain	33(16.3)	38(18.8)	60(29.7)	42(20.8)	29(14.4)	2.95+1.35 Median (3.0) IQR (2)
Coughing up blood	41(20.3)	50(24.8)	56(27.7)	34(16.8)	21(10.4)	3.21+1.34 Median (3.0) IQR (2)
An ache or pain when breathing	41(20.3)	57(28.2)	53(26.2)	34(16.8)	17(8.4)	3.26+1.33 Median (3.0) IQR (2)
Loss of appetite	38(18.8)	41(20.3)	56(27.7)	33(16.3)	34(16.8)	3.08+1.33 Median (3.0) IQR (2)
Painful cough	51(25.2)	43(21.3)	48(23.8)	35(17.3)	25(12.4)	3.24+1.41 Median (3.0) IQR (3)
Changes in the shape of your fingers or nails	36(17.8)	37(18.3)	67(33.2)	34(16.8)	28(13.9)	3.06+1.30 Median (3.0) IQR (2)
Developing an unexplained loud, high-pitched sound when breathing	40(19.8)	36(17.8)	72(35.6)	28(13.9)	26(12.9)	3.16+1.27 Median (3.0) IQR (2)
Worsening or change in an existing cough	47(23.3)	40(19.8)	61(30.2)	35(17.3)	19(9.4)	3.22+1.36 Median (3.0) IQR (2)

**Table 3:** Frequencies of undergraduates correct answers toward Knowledge of awareness of LC symptoms (n=202).

Variables Non-respiratory symptoms	Pharmacy n(%)	Nursing n(%)	Total n(%)
Unexplained weight loss	41(44.1)	37(33.9)	78(38.6)
Persistent tiredness or lack of energy	47(50.5)	46(42.2)	93(46.0)
Persistent shoulder pain	33(35.5)	38(34.9)	71(35.1)
Loss of appetite	41(44.1)	38(34.9)	79(39.1)
Changes in the shape of fingers or nails	28(30.1)	45(41.3)	73(36.1)
Developing an unexplained loud, high-pitched sound when breathing	37(39.8)	39(35.8)	76(37.6)
**Respiratory symptoms**			
Persistent chest infection	38(40.9)	36(33.0)	74(36.6)
A cough that does not go away for two or three weeks	38(40.9)	38(34.9)	76(37.6)
Persistent shortness of breath	48(51.6)	49(45.0)	97(48.0)
Persistent chest pain	47(50.5)	40(36.7)	87(43.1)
Coughing up blood	52(55.9)	39(35.8)	91(45.0)
An ache or pain when breathing	56(60.2)	42(38.5)	98(48.5)
Painful cough	49(52.7)	45(41.3)	94(46.5)
Worsening or change in an existing cough	46(49.5)	41(37.6)	87(43.1)

**Table 4:** Association between Awareness of, undergraduates and their characters.

Characteristics	Awareness			p-value
	Good	Moderate	Poor	
	n(%)	n(%)	n(%)	
**Gender**				<0.001
Male	68(42.8)	47(29.6)	44(27.7)	
Female	32(74.4)	02(4.7)	09(20.9)	
**Course of study**				0.032
Pharmacy	55(59.1)	20(21.5)	18(19.4)	
Nursing	45(41.3)	29(26.6)	35(32.1)	
**Year of Study**				<0.001
Third	06(35.3)	08(47.1)	3(17.6)	
Fourth	18(47.4)	10(26.3)	10(26.3)	
Fifth	44(41.1)	29(27.1)	34(31.8)	
Final	32(80.0)	02(5.0)	06(15.0)	
**Have you ever smoked cigarettes?**				0.002
Never smoked	86(57.3)	31(20.7)	33(22.0)	
Former smoker	06(24.0)	11(44.0)	08(32.0)	
Current smoker	08(29.6)	07(25.9)	12(44.4)	
**Have you ever smoked water pipes/shisha?**				0.035
Never smoked	92(53.8)	38(22.2)	41(24.0)	
Former smoker	06(24.0)	08(32.0)	11(44.0)	
Current smoker	02(33.3)	03(50.0)	1(16.7)	
**Do you have any chronic diseases?**				0.163
Yes	17(65.4)	03(11.5)	06(23.1)	
No	83(47.2)	46(26.1)	47(26.7)	
**Do you know someone diagnosed with cancer?**				0.089
**Yes**	33(61.1)	08(14.8)	13(24.1)	
**No**	67(45.3)	41(27.7)	40(27.0)	

**Table 5:** Regression results of knowledge of awareness with some demographic features of students.

Model	Unstandardized Coefficients		t	*p-value*	95.0% CI for B	
	B	Std. Error			Lower Bound	Upper Bound
(Constant)	2.163	3.373	.641	.522	-4.489	8.815
Age	.164	.184	.896	.372	-.198	.527
Gender	2.823	1.065	2.652	**.009**	.723	4.923
Marital status	-5.149	1.243	-4.142	**<.001**	-7.601	-2.698
Your course of study	-.827	.794	-1.042	.299	-2.393	.739
Year of Study	.374	.400	.936	.351	-.415	1.163
Do you have any chronic diseases?	3.292	1.015	3.243	**.001**	1.290	5.295
Do you know someone diagnosed with cancer	.116	.767	.151	.880	-1.398	1.630
Have you ever smoked cigarettes	-.659	.607	-1.086	.279	-1.855	.538
Have you ever smoked water pipe tobacco\Shisha	-.753	.930	-.810	.419	-2.587	1.081
